# Uniparental disomy and prenatal phenotype: Two case reports and review

**DOI:** 10.1097/MD.0000000000008474

**Published:** 2017-11-10

**Authors:** Xiaofei Li, Yan Liu, Song Yue, Li Wang, Tiejuan Zhang, Cuixia Guo, Wenjie Hu, Karl-Oliver Kagan, Qingqing Wu

**Affiliations:** a Department of Ultrasound; b Department of Obstetrics, Beijing Obstetrics and Gynecology Hospital, Capital Medical University, Beijing, China; c Department of Obstetrics and Gynecology, University of Tübingen, Tübingen, Germany.

**Keywords:** phenotype-genotype, prenatal ultrasound, single-nucleotide polymorphism-based array, small supernumerary marker chromosomes, uniparental disomy

## Abstract

**Rationale:**

Uniparental disomy (UPD) gives a description of the inheritance of both homologues of a chromosome pair from the same parent. The consequences of UPD depend on the specific chromosome/segment involved and its parental origin.

**Patient concerns:**

We report prenatal phenotypes of 2 rare cases of UPD.

**Diagnoses:**

The prenatal phenotype of case 1 included sonographic markers such as enlarged nuchal translucency (NT), absent nasal bone, short femur and humerus length, and several structural malformations involving Dandy–Walker malformation and congenital heart defects. The prenatal phenotype of Case 2 are sonographic markers, including enlarged NT, thickened nuchal fold, ascites, and polyhydramnios without apparent structural malformations.

**Interventions:**

Conventional G-band karyotype appears normal in case 1, while it shows normal chromosomes with a small supernumerary marker chromosome (sSMC) in case 2. Genetic etiology was left unknown until single-nucleotide polymorphism-based array (SNP-array) was performed, and segmental paternal UPD 22 was identified in case 1 and segmental paternal UPD 14 was found in case 2.

**Outcomes:**

The parents of case 1 chose termination of pregnancy. The neonate of case 2 was born prematurely with a bellshaped small thorax and died within a day.

**Lessons:**

UPD cases are rare and the phenotypes are different, which depend on the origin and affected chromosomal part. If a fetus shows multiple anomalies that cannot be attributed to a common aneuploidy or a genetic syndrome, or manifests some features possibly related to an UPD syndrome, such as detection of sSMC, SNP-array should be considered.

## 1 Introduction

Uniparental disomy (UPD) is defined as 2 homologous chromosomes, or segments of chromosomes, originated from the same parent.^[1]^ UPD can either be congenital or be acquired, and the latter is usually acquired during tumor initiation and progression.^[2]^ UPD may comprise the whole chromosome, or just part of it (segmental UPD). Isodisomy is presented as 2 copies from the same chromosome of a parent, and heterodisomy is presented as 1 copy of each of the 2 homologues from the same parent.

Unlike genetic diseases such as microdeletion and microduplication, most of the UPDs are not inherited from the genetic defects of the parents, but are related to abnormalities formed during meiosis, fertilization, and mitosis. There are 5 different mechanisms that explain the etiology of UPD,^[3]^ including Trisomic rescue; Nullisomic gamete complementation; Monosomic rescue; Mitotic aberrations; and Structural chromosome aberrations. If prenatal genetic analysis reveals a homologous robertsonian translocation, the risk of fetal UPD is very high,^[4]^ and a marker chromosome or chromosome aneuploidy can be associated with UPD.

There have been no precise data on the incidence of chromosome-specific UPDs. When assuming that for a particular chromosome, the frequency of disomy and nullisomy of each sperm is of 0.10% in males, which is 5 times higher in females, UPD for a particular chromosome might be expected to occur by gamete complementation at a frequency of 1/100,000 births and for any of the 22 autosomes in approximately 1/5000 births.^[5]^ As far as we know, all of the reported paternal UPD 22 cases were diagnosed after birth. Although several paternal UPD 14 cases were diagnosed prenatally, none of them was suspected by untypical prenatal ultrasound findings mall supernumerary marker chromosomes.

In this study, 2 UPD cases were diagnosed perinatally by performing single-nucleotide polymorphism-based array (SNP-array). One of them was segmental paternal UPD 22 with apparent phenotypes, while another one was segmental paternal UPD 14 with sSMC demonstrating some nonspecific features without apparent structural abnormalities prenatally. The cases of paternal UPD 22 and paternal UPD 14 from PubMed database were reviewed.

## 2 Case report

We report 2 rare cases of UPD. Informed consents were obtained from these patients. The case reports were approved by the Ethics Committees of Beijing Obstetrics and Gynecology Hospital, Capital Medical University.

**Figure 1:**
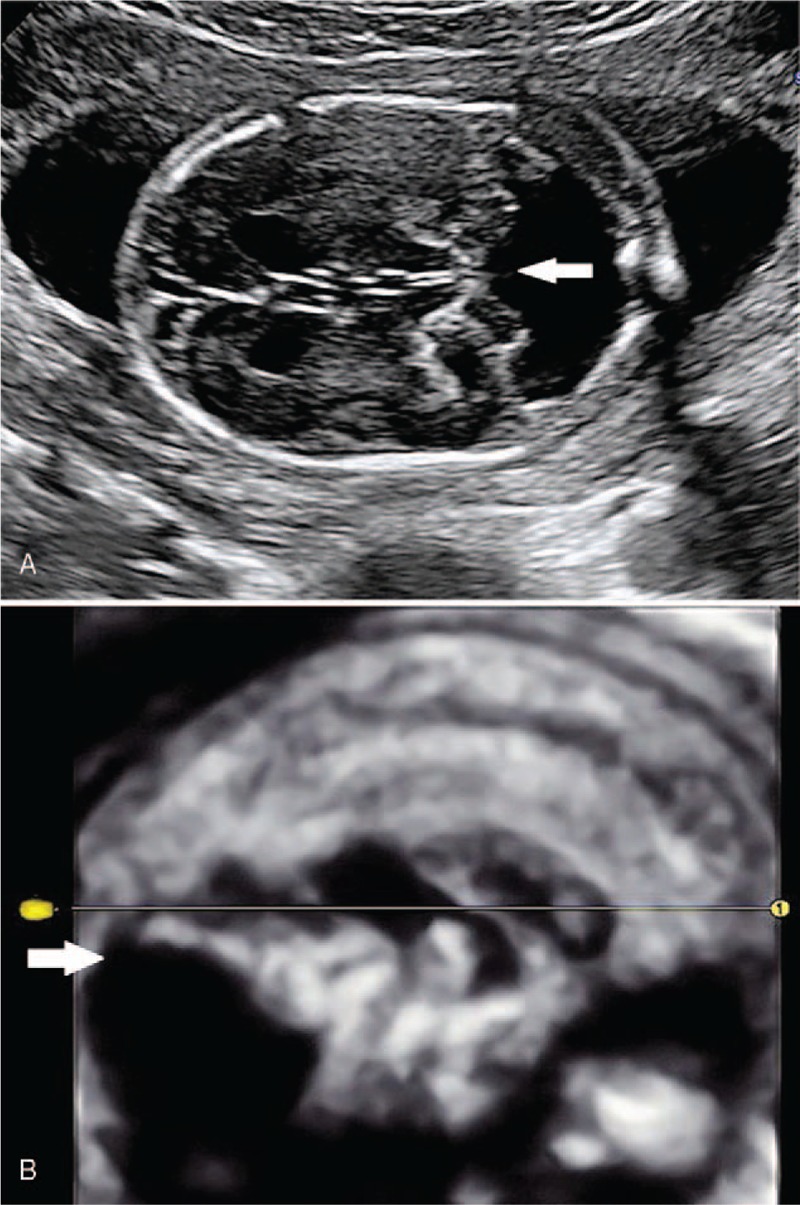
The sonographic examination of Case 1 at 21 weeks’ gestational age. (A) Transverse scan through the cerebellum showed Dandy–Walker malformation (white arrowhead). (B) 3D ultrasound rendering of the incranial structures showed the absence of cerebellar vermis and elevation of tentorium cerebelli (white arrowhead).

**Figure 2:**
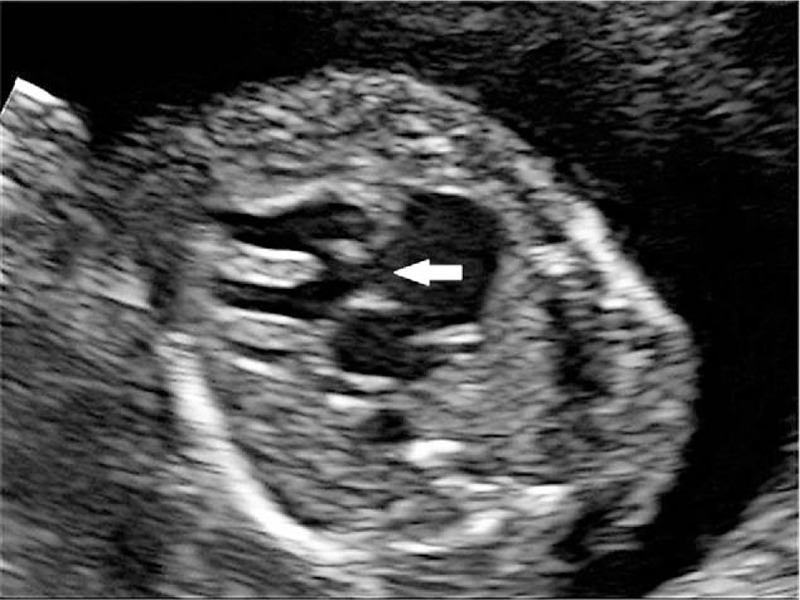
The sonographic examination of case 1 at 21 weeks’ gestational age. Four-chamber view of the fetal heart showed atrioventricular septal defect (white arrowhead).

Case 1 is a fetus of a 28-year-old woman, primigravida, who conceived naturally. The previous medical history of the woman was normal, and there was no family history of congenital anomalies. The mother did not have abnormal signs and symptoms, and she denied teratogenic exposure. The serologic test for TORCH infection diseases (including Toxoplasmosis, Rubella, Cytomegalovirus, Herpes simplex virus) was negative. The sonographic screening at 13 weeks’ gestation revealed enlarged nuchal translucency thickness (NT), increased intracranial translucency (IT) diameter, absent nasal bone. Ventricular septal defect was suspected as well. Follow-up scannings were performed at 16 and 21 gestation weeks. Major anomalies, Dandy–Walker malformation (Fig. 1), congenital heart defects [atrioventricular septal defect (Fig. 2), double outlet right ventricle and mild pulmonary artery stenosis] were identified. Other sonographic markers and other minor anomalies were detected as well, including absent nasal bone, short femur and humerus length, arachnoid cyst, small stomach bubble, and irregular spinal alignment.

**Figure 3:**
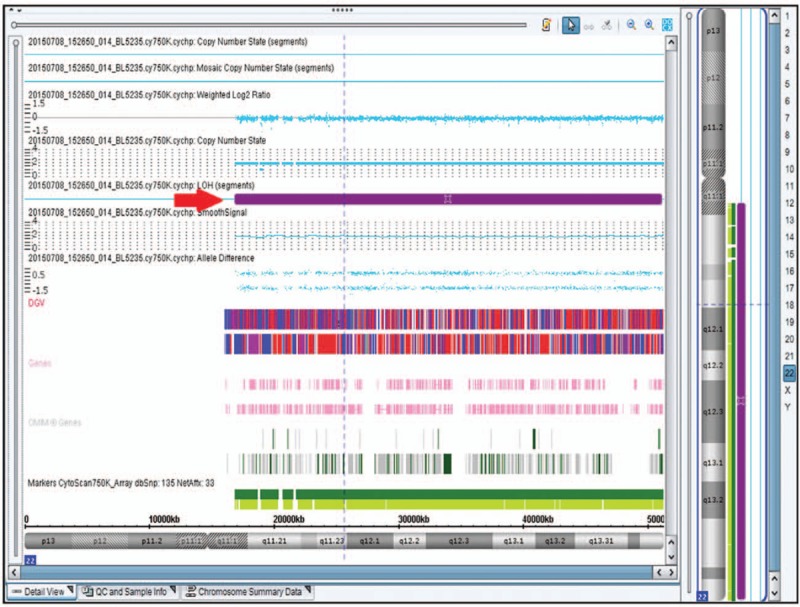
SNP-array (Affymetrix CytoScan 750K Array) report of case 1. No copy number variations were detected but loss of heterozygosity on long arm of chromosome 22 (red arrowhead).

Noninvasive prenatal test showed a low risk of trisomy 13, 18, 21 and common sex chromosome aneuploidies. Subsequently amniocentesis was carried out to further identify chromosomal anomalies. Conventional G-band karyotype and fluorescence in situ hybridization (FISH) analysis revealed normal karyotypes. Then, chromosomal microarray analysis (CMA) using SNP-array (Affymetrix CytoScan 750K Array, Santa Clara, California) was performed, which finally demonstrated no copy number variations but loss of heterozygosity on long arm of chromosome 22 [ISCN: arr(hg19) 22q11.1q13.33(16,888,899–51,157,531)x2 hmz] (Fig. 3).

Considering the major structural and genotype anomalies, the parents decided to terminate the pregnancy at 22 weeks’ gestation. This woman delivered a healthy child in the next year.

**Figure 4:**
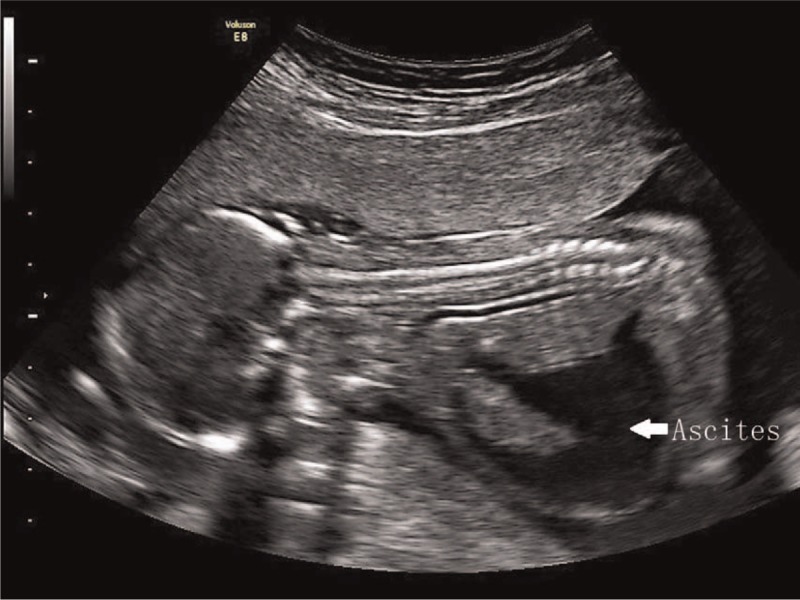
The sonographic examination of case 2 at 16 weeks’ gestation. No structural abnormalities were found, but unexplained ascites (white arrowhead) and thickened nuchal fold were identified.

Case 2 is a fetus of a 27-year-old woman, primigravida, who conceived naturally. Her medical and family history was normal. Teratogenic exposure was denied. No complaint of specific symptoms by the mother during the pregnancy. The first-trimester sonographic screening at 13 weeks’ gestation revealed isolated enlarged NT. Further detailed sonographic examination was carry out at 17 and 21 weeks’ gestation, and no structural anomalies were found, while thickened nuchal fold and ascites (Fig. 4) were detected. Then, ascites was reduced gradually, but polyhydramnios appeared in the follow-up scannings between 27 and 31 weeks’ gestation.

**Figure 5:**
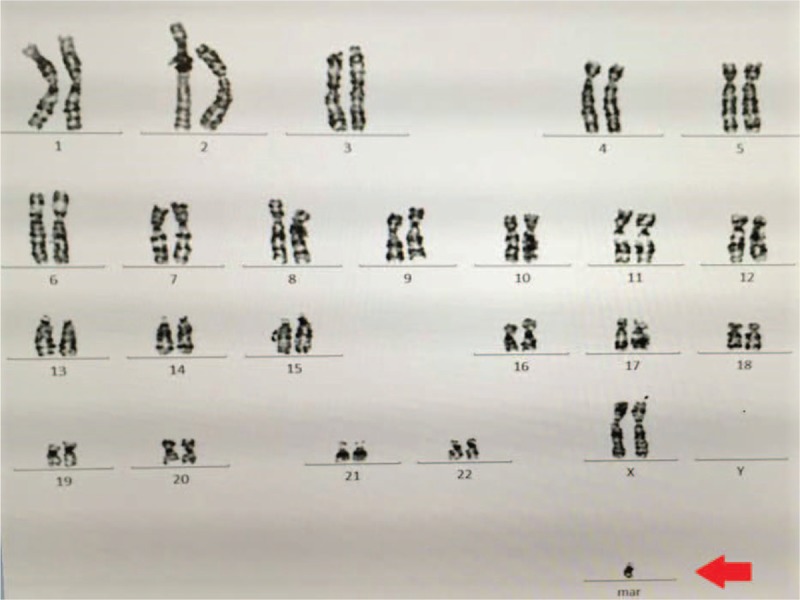
Conventional G-band karyotype analysis report of Case 2 revealed 46 normal chromosomes with a small supernumerary marker chromosome (sSMC) (red arrowhead).

**Figure 6:**
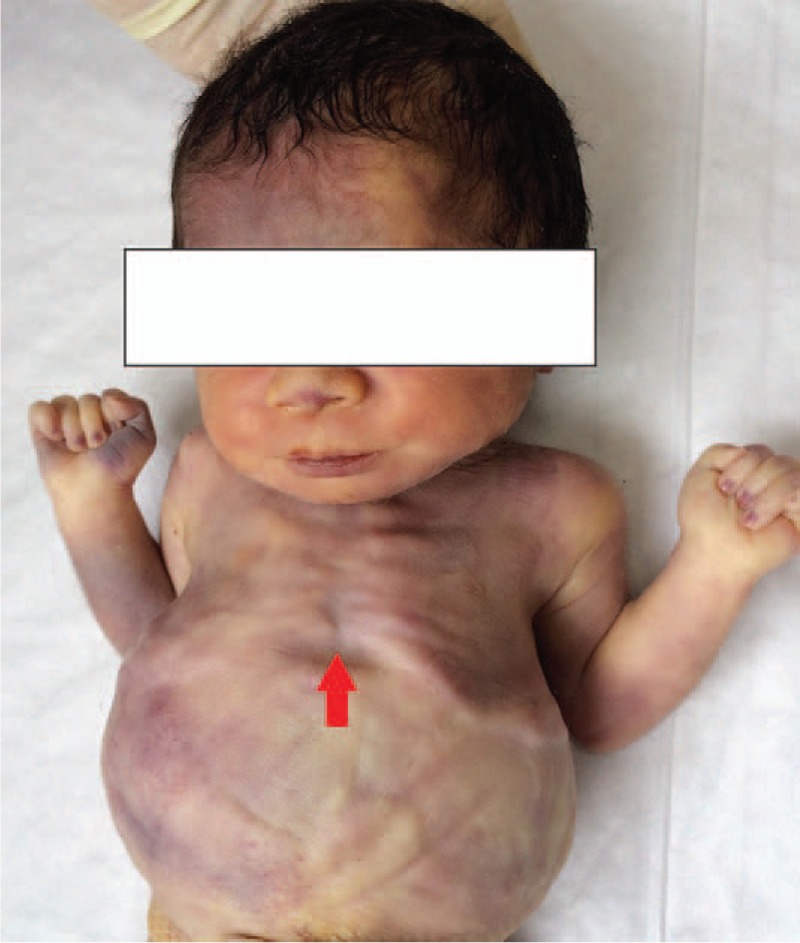
Gross inspection of case 2 showed a bell-shaped small thorax (red arrowhead).

**Figure 7:**
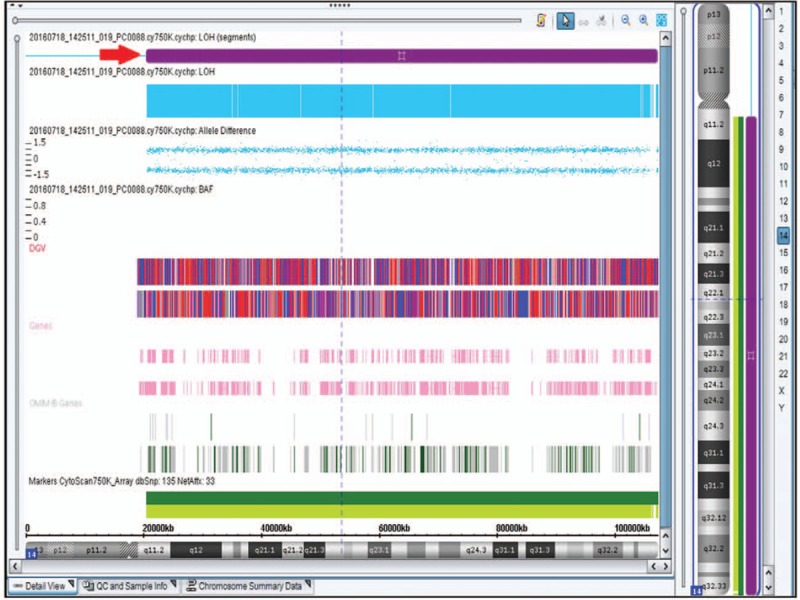
SNP-array (Affymetrix CytoScan 750K Array) report of case 2. No copy number variations were detected but loss of heterozygosity on long arm of chromosome 14 (red arrowhead).

This fetus received amniocentesis for the purpose of reducing amniotic fluid volume, checking the ascites and performing prenatal diagnostic testing. Conventional G-band karyotype analysis revealed 46 normal chromosomes with a small supernumerary marker chromosome (sSMC) (Fig. 5). The results of amniotic test for TORCH infection and fetal ascites culture and biochemical examination were negative. Considering no major karyotypic or structural anomalies were identified so far, the parents decided to carry on the pregnancy. The baby was born prematurely at 33 weeks’ gestation with a bell-shaped small thorax (Fig. 6) and died within a day. CMA using SNP-array (Affymetrix CytoScan 750K Array) was then performed, no copy number variations were detected, but the long arm of chromosome 14 was detected by loss of heterozygosity [ISCN:arr[hg19]14q11.2q32.33(20,520,197–107,279,475)x2 hmz] (Fig. 7).

Karyotype analysis of these 2 parents showed that they are normal individuals. Linkage analysis by polymerase chain reaction (PCR)-based Sequence-Tagged Site (STS) marker showed that the segmental isodisomy of both cases was of paternal origin.

## 3 Discussion

Chromosomal abnormalities are associated with perinatal death and birth defects. The detection rate of congenital malformations associated with chromosomal anomalies by G-band karyotype analysis is 9 ∼ 35%,^[6–8]^ depending on whether the anomaly is isolated or multiple. SNP-array analysis is a kind of microarray technologies. It has a higher resolution up to tens of KB that can be used to examine patients’ genome for detecting additions or losses of genetic material that are too small to be detectable by G-band karyotype analysis.^[9,10]^ Therefore, SNP-array analysis could detect about 10% genetic abnormalities additionally.^[9,11]^ Furthermore, SNP-array is able to identify UPD.^[9]^

The phenotypes of UPD range from unapparent to typical autosomal-recessive disease or syndromic imprinting disorder, depending on the parental origin and the specific chromosome or segment involved.^[12,13]^

Case 1 was a fetus with apparent prenatal phenotypes, including several sonographic markers such as enlarged NT, increased IT diameter (an important risk factor for cystic posterior fossa malformations and chromosomal aberrations),^[14]^ absent nasal bone, short femur and humerus length, and multiple structural abnormalities involving Dandy–Walker malformation and congenital heart defects. Our group has found that sonographic phenotypes are related to genotypes by using CMA.^[15–19]^ Considering this, series of genetic analyses were performed in order. G-band karyotype analysis and FISH were performed to exclude aneuploidy. FISH also was used for well-known syndromes confirmation, such as Angelman syndrome and DiGeorge syndrome. Then, SNP-array was performed.

To our knowledge, there have been only 3 cases of paternal UPD 22 reported (Table 1),^[20–22]^ two of them phenotypically normal, and both of them and their father have robertsonian translocation.^[20,21]^ The third one had metachromatic leukodystrophy,^[22]^ which is an autosomal recessive disease caused by defects of a protein coding gene *ARSA* (OMIM ID: ^∗^607574). His father and sister were detected as carriers of this gene, while his mother did not have mutated allele.^[22]^ Therefore, this patient developed the disease by uniparental isodisomy. But all of these 3 cases are different from our case, as they are all diagnosed in adulthood, while our case was diagnosed prenatally.

**Table 1:**
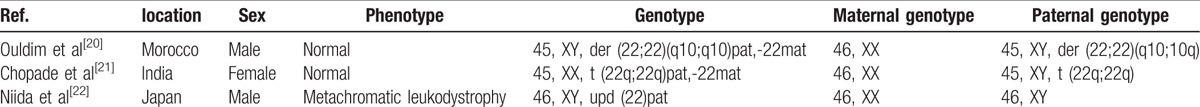
General information, phenotypes, and genotypes of fetal paternal UPD 22 cases.

The reason why our first case was associated with apparent phenotypes still remains unclear. We suppose that there might be 2 possibilities. On the one hand, the phenotype may be related to autosomal recessive genetic disease. There are several genes located on 22q, which are related to Dandy–Walker malformation and congenital heart defects according to omim.org, including TUBGCP6 (OMIM ID:^∗^251270), GOMBO (OMIM ID: ^∗^233270), LARGE (OMIM ID: ^∗^608840 or ^∗^613154), SLC25A1 (OMIM ID: ^∗^615182), PI4KA (OMIM ID: ^∗^616531), and *CDC45L* gene (OMIM ID: ^∗^617063). On the other hand, disturbed genomic imprinting is the biological base of UPD. There are 3 imprinting genes located on 22q according to www.geneimprint.com/site/genes-by-species.Homo+sapiens: DGCR6L, DGCR6,^[23]^ and FLJ20464.^[24]^ The first 2 genes are related to DiGeorge syndrome, which is frequently associated with congenital heart defects, and that might be the reason for our case.

Paternal UPD 14 and related conditions are also known as Kagama–Ogata syndrome. There has been over 20 case reports about paternal UPD 14.^[3,12,13,25–29]^ Unique facial appearance, specific configuration of the thoracal ribs (“coat-hanger sign”), bell-shaped thorax, polyhydramnios, intrauterine growth restriction, and cardiomyopathy are cardinal features prenatally. Most cases die shortly after birth or in infancy. Segmental paternal UPD, epimutations, and microdeletions affecting the IG-DMR or the MEG3-DMR of maternal origin are necessary and sufficient for the characteristic.^[25]^

Although our second case manifested the typical features of paternal UPD 14, its genotype was atypical---paternal UPD 14 complicated with sSMC. Some researchers have already found the relationship between UPD and sSMC. Liehr et al ^[30]^ had reported 46 cases of sSMC with UPD, most of them (87%) were of maternal origin. Only 1 case was sSMC with paternal UPD14. Some were pathogenic (30%) being correlated with specific syndromes,^[31]^ but most of them were carriers and clinically asymptomatic. In our second case, although the origin of sSMC was unknown, the phenotype was consitient with Kagama–Ogata syndrome due to paternal UPD14. Therefore, if a fetus demonstrates typical features of UPD syndrome, especially when the G-band karyotype analysis shows sSMC, the diagnosis of UPD syndrome should be considered.

## 4 Conclusion

We reported 2 cases of UPD, one was segmental paternal UPD 22 with apparent phenotypes, another one was segmental paternal UPD 14 with sSMC, manifesting some nonspecific features without apparent structural abnormalities prenatally. Both cases were rare, and the genetic abnormalities were unknown until SNP-array was performed. Therefore, if a fetus shows multiple sonographic markers and structural abnormalities that are not specific to a common aneuploidy or a well-known genetic syndrome, or if G-band karyotype shows sSMC and typical features of a known UPD syndrome were detected, SNP-array should be considered.
